# Presence of the Weakly Pathogenic *Fusarium poae* in the Fusarium Head Blight Disease Complex Hampers Biocontrol and Chemical Control of the Virulent *Fusarium graminearum* Pathogen

**DOI:** 10.3389/fpls.2021.641890

**Published:** 2021-02-17

**Authors:** Jiang Tan, Noémie De Zutter, Sarah De Saeger, Marthe De Boevre, Trang Minh Tran, Theo van der Lee, Cees Waalwijk, Anne Willems, Peter Vandamme, Maarten Ameye, Kris Audenaert

**Affiliations:** ^1^Laboratory of Applied Mycology and Phenomics, Department of Plants and Crops, Faculty of Bioscience Engineering, Ghent University, Ghent, Belgium; ^2^Centre of Excellence in Mycotoxicology and Public Health, Department of Bioanalysis, Faculty of Pharmaceutical Sciences, Ghent University, Ghent, Belgium; ^3^Business Unit Biointeractions and Plant Health, Wageningen University and Research, Wageningen, Netherlands; ^4^Laboratory of Microbiology, Department of Biochemistry and Microbiology, Faculty of Sciences, Ghent University, Ghent, Belgium

**Keywords:** *Fusarium*, actinobacteria, pathobiome, biocontrol, multispectral imaging

## Abstract

Fusarium head blight (FHB) in wheat (*Triticum aestivum* L.) is caused by a consortium of mutually interacting Fusarium species. In the field, the weakly pathogenic *F. poae* often thrives on the infection sites of the virulent *F. graminearum*. In this ecological context, we investigated the efficacy of chemical and biocontrol agents against *F. graminearum* in wheat ears. For this purpose, one fungicide comprising prothioconazole + spiroxamine and two bacterial biocontrol strains, *Streptomyces rimosus* LMG 19352 and *Rhodococcus* sp. R-43120 were tested for their efficacy to reduce FHB symptoms and mycotoxin (deoxynivalenol, DON) production by *F. graminearum* in presence or absence of *F. poae*. Results showed that the fungicide and both actinobacterial strains reduced FHB symptoms and concomitant DON levels in wheat ears inoculated with *F. graminearum*. Where *Streptomyces rimosus* appeared to have direct antagonistic effects, *Rhodococcus* and the fungicide mediated suppression of *F. graminearum* was linked to the archetypal salicylic acid and jasmonic acid defense pathways that involve the activation of *LOX1*, *LOX2* and *ICS*. Remarkably, this chemical- and biocontrol efficacy was significantly reduced when *F. poae* was co-inoculated with *F. graminearum*. This reduced efficacy was linked to a suppression of the plant’s intrinsic defense system and increased levels of DON. In conclusion, our study shows that control strategies against the virulent *F. graminearum* in the disease complex causing FHB are hampered by the presence of the weakly pathogenic *F. poae*. This study provides generic insights in the complexity of control strategies against plant diseases caused by multiple pathogens.

## Introduction

The need for resilient crops to cope with pests and diseases gains importance in a changing climate (Bebber et al., 2013). Driven by globalization and trade, pathogen pressure has increased due to emergent plant pathogens which threaten our agro-ecosystems (Corredor-Moreno and Saunders, 2020). In the context of global warming, the disease Fusarium head blight (FHB) is of particular interest as it is considered an increasing problem for cereal crops (Juroszek and von Tiedemann, 2013). The disease is highly dynamic and caused by a complex of different *Fusarium* species that can each respond differently to the changing climate (Yli-Mattila, 2010; Vaughan et al., 2016).

Within this species complex causing FHB, the hemi-biotrophic ascomycete fungus *Fusarium graminearum* is the predominant species worldwide. *F. graminearum* deploys a hemi-biotrophic strategy to infect plants and produces amongst others the trichothecene mycotoxin deoxynivalenol (DON) as a virulence factor during infection (Audenaert et al., 2014; Ameye et al., 2015). This molecule is the main virulence factor allowing *F. graminearum* to colonize the rachilla and rachis of the spikelets and ear, respectively by interacting with the primary N- and C-metabolism of the plant and with archetypal plant defense pathways, such as the oxidative burst and the salicylic acid (SA) and jasmonic acid (JA) directed signaling networks (Gardiner et al., 2009; Seifi et al., 2013; Audenaert et al., 2014; Ameye et al., 2015).

However, *F. graminearum* is only one member of the species complex causing FHB and the composition of the complex is geo-dependent. In addition, pathogens of secondary importance such as *F. asiaticum* and *F. boothii* emerge occasionally or gain in importance due to environmental or agronomic factors (Parry et al., 1995). In general, *F. graminearum*, *F. culmorum*, *F. avenaceum*, and *F. poae* are considered as the predominant species associated with FHB worldwide (Ioos et al., 2005; Xu et al., 2005). Despite the presence of these species in diseased kernels, only *F. graminearum* and *F. culmorum* are considered highly pathogenic, while the other species are less aggressive on wheat (*Triticum aestivum* L.). For a long time it was thought that they may infect, but do not proliferate, grow superficially and thrive primarily at the sites of inoculation (Stack and McMullen, 1985; Brennan et al., 2003). However, novel insights have shown that the multispecies interaction goes beyond a merely ‘one plus one is two’ theorem.

Recently, we showed that symptom development upon infection by two members of the FHB disease, *F. graminearum* and *F. poae*, is determined by timing of infection. Pre-inoculation of wheat ears with the weakly pathogenic *F. poae* prior to *F. graminearum* resulted in a disappearance of FHB symptoms and a reduction of mycotoxin levels compared to a single *F. graminearum* infection (Tan et al., 2020). In contrast, in these interactions, *F. poae* exhibited increased growth demonstrating that this weak pathogen takes advantage from its co-occurrence with *F. graminearum*. Moreover, *F. poae* was shown to asymptomatically induce SA and JA related defenses which hamper a later infection by *F. graminearum* (Ameye et al., 2015; Tan et al., 2020).

The multi-species nature of this disease also influences its control. From a crop protection point of view, the multispecies composition implies that different species of the FHB complex might react differently upon exposure to fungicides or biocontrol agents. In this regard, it has been demonstrated that especially weak pathogens such as *F. langsethiae, F. avenaceum*, and *F. poae* are more resistant to the ergosterol biosynthesis inhibiting azole fungicides than *F. graminearum* (Tini et al., 2020). Upon treatment in the field with azole fungicides, this results in an increased share of *F. poae* in the FHB population at the expense of *F. graminearum* (Audenaert et al., 2011). In view of these issues, authorities aspire to reduce the dependency, risk and use of fungicides and opt for alternative control measures.

Anticipating this paradigm shift, research has turned to the exploration of agricultural inoculants composed of bioactive bacterial isolates that exhibit significant biocontrol activities and new innovative strategies to apply them in the field. Actinobacteria have been described to exhibit a wide variety of potential biocontrol activities such as mycoparasitism, production of antibiotics and production of cell wall degrading enzymes (Palaniyandi et al., 2013). In addition, they combine these traits with a huge metabolic versatility, filamentous growth, spore formation capability and an omnipresence in the agro-ecological relevant niche of the soil (Newitt et al., 2019). Besides these direct interactions with the pathogen, evidence is accumulating that several Actinobacteria can also interact with the plant defense system. For example, it is known that several Actinobacteria can tailor their secondary metabolite production upon exposure to plant defense hormones (Van der Meij et al., 2018). Finally, they can prime the SA- and/or JA/ethylene dependent defense pathways in plants (Conn et al., 2008; Kurth et al., 2014; Vergnes et al., 2020). However, these defense-related pathways form an intricate signaling network with synergistic as well as antagonistic interactions, which adds another layer of complexity on the efficacy of biocontrol agents in plant disease management (Robert-Seilaniantz et al., 2011).

In the present paper, we investigated the impact of two promising Actinobacteria strains for their effectiveness to reduce FHB and concomitant mycotoxins in wheat. We unraveled their mode of action and showed that their capacity to reduce FHB symptoms caused by *F. graminearum* depends on the presence or absence of *F. poae* in the ear. In addition, this change in biocontrol capacity is linked to the archetypal SA- and JA- dependent plant defense pathways and the capacity to glucosylate DON in the ear.

## Materials and Methods

### *Fusarium* Strains: Growth and Production of Conidia

The *Fusarium* strains used in present study were the GFP transformants of *F. graminearum* PH-1 (Trail and Common, 2000) and *F. poae* 2516 (Vanheule et al., 2016) that have been previously introduced in the study by Tan et al. (2020). The GFP transformants of *F. graminearum* strain PH-1 or *F. poae* 2516 were grown on potato dextrose agar (PDA, Sigma-Aldrich) for 7 days at 21°C under a regime of 12 h of dark and 12 h of combined UVA and UVC light (2 × TUV 8W T5 and 1 × TL 8W BLB; Philips, Eindhoven, Netherlands). Conidia were harvested by adding a solution of sterile 0.01% (v/v) Tween 80 to the PDA plates and rubbing the mycelium with a Drigalski spatula. Four layers of sterile miracloth were used to filter the conidia and remove the mycelium. The suspension was adjusted to a final concentration of 1 × 10^6^ conidia mL^–1^. This suspension was used for inoculations on both wheat leaves and ears.

### Actinobacterial Strains

The actinobacterial strains comprising both *Streptomyces* and *Rhodococcus* species are listed in Table 1. The bacterial strains were grown from the –80°C glycerol stock for 3 days at 25°C in 5 mL Tryptic Soy Broth (TSB, Sigma-Aldrich) at 200 rpm. Subsequently, the bacteria were washed twice with PBS (Phosphate buffered saline) to be used in the biocontrol assays.

**TABLE 1:** Actinobacterial strains for biocontrol testing.

**Number**	**Strain**	**Reference/depositor**
1	*R. pyridinivorans* SB3094	Dueholm et al., 2014
2	*R. erythropolis BD2*	Stecker et al., 2003
3	*R. erythropolis SQ1*	Quan and Dabbs, 1993
4	*R. rodnii* LMG5362	Pachebat et al., 2013
5	*R. defluvii* Ca11^*T*^	Kämpfer et al., 2014
6	*R. jostii RHA1*	McLeod et al., 2006
7	*R. corynebacteriodes JK1*	Stamler et al., 2015
8	*R. fascians SR13*	Stamler et al., 2015
9	*R. fascians D188-5*	Desomer et al., 1988
10	*R. globerulus* DSM43954	DSMZ-German Collection of Microorganisms and Cell Cultures
11	*R. ruber* DSM43338	DSMZ-German Collection of Microorganisms and Cell Cultures
12	*Rhodococcus sp.* R-43120	Anne Willems, Research collection
13	*S. turgidiscabies 90*	Francis et al., 2015
14	*S. turgidiscabies 97*	Francis et al., 2015
15	*S. cacaoi sub. asoensis* K234	Harkai et al., 2016
16	*S. rimosus K145*	Harkai et al., 2016
17	*S. rimosus K189*	Harkai et al., 2016
18	*S. humiferus B5*	Vanhoutte et al., unpublished data
19	*S. humiferus B6*	Vanhoutte et al., unpublished data
20	*S. rimosus* subsp. *rimosus LMG 19352*	1984, National Collection of Industrial, Food and Marine Bacteria, Ltd. (NCIMB) < - 1951, M. Lumb

### *In vitro* Antagonism

In an *in vitro* plate assay, the antagonism of *S. rimosus* LMG 19352 and *Rhodococcus* sp. R-43120 toward *F. graminearum* and *F. poae* was assessed. Bacterial suspensions and fungal conidia were obtained as previously described. Five μL of a conidial suspension at 1 × 10^6^ mL^–1^ of either strains was inoculated in the center of a petri dish. Subsequently, four spots of 5 μL of the actinobacterial suspensions were applied in a square at 3 cm from the fungal inoculation spot. Control plates were inoculated with either of both fungi (*F. graminearum* or *F. poae*). Fungal diameter was assessed daily till 5 days after inoculation.

### Plant Materials, Detached Leaf Assay and Whole Plant Ear Assay

The biocontrol capabilities of the actinobacterial strains were tested in a high throughput detached leaf assay. For this assay, spring wheat cultivar (cv) Tybalt was grown in pots (4.5-cm diameter × 6.5-cm height) in a growth chamber (21°C, 16 h: 8 h, light: dark) for 10 days. Leaf segments of 4 cm were cut from the tip of the oldest leaves of 10 days old seedlings. These leaves were placed on their abaxial surface in Petri dishes containing 0.5% (w/v) water agar amended with 40 mg L^–1^ benzimidazole. GFP tagged *F. graminearum* PH-1 was applied as a conidial suspension of 2.5 μL which was deposited in the center of the leaf segment which had been wounded by using a sterile inoculation needle prior to the inoculation (Ameye et al., 2015). Subsequently, a droplet of 2.5 μL of a bacterial suspension containing 1 × 10^8^ CFUs per mL was applied on the same spot as the conidial suspension. The disease progress was assessed using a multispectral imaging approach (see below).

Based on the detached leaf assay, the two best performing biocontrol strains, i.e., *Streptomyces rimosus* LMG 19352 and *Rhodococcus* sp. R-43120 were used to assess their impact on two members of the FHB disease complex. These experiments were done using a wheat ear bioassay on intact plants previously optimized by Tan et al. (2020). For the wheat ear bioassay, about 10 spring wheat cv Tybalt plants were grown per pot (15-cm diameter × 30-cm height) under glasshouse conditions till Zadok’s Growth Scale (GS) 65 (Zadoks et al., 1974). In total 6 replicates were included per treatment and the experiment was repeated once in time. For each treatment, the 6 replicates always originated from different pots.

To assess the impact of *Streptomyces rimosus* strain LMG 19352, *Rhodococcus* sp. strain R-43120 and the fungicide cocktail prothioconazole + spiroxamine on the infection of *F. graminearum* and/or *F. poae*, wheat ears were infected with *F. graminearum* (g + w), *F. poae* (p + w) or co-inoculated with both pathogens (pg + w). The biocontrol treatments for *F. poae* comprised *F. poae* + LMG 19352 (p + b1), R-43120 (p + b2) and fungicide (p + f). Similar treatments with *F. graminearum* resulted in treatment labels (g + b1), (g + b2), and (g + f). Simultaneously infected *F. poae* and *F. graminearum* ears comprised (pg + w), (pg + b1), (pg + b2), and (pg + f). Control treatments to assess the phytotoxicity of the applied biocontrol strains or the fungicide consisted of (w + b1), (w + b2), and (w + f).

### Disease Progress: Visual Assessment and Multispectral Phenomics

Visual scoring assessment of ears inoculation was performed at 7 days after inoculation (dai) by using a five class disease index, where Level 1 = no symptoms visible, Level 2 = one spikelet with symptoms, Level 3 = two spikelets with symptoms, and Level 4 = all of the spikelets from the inoculation site upwards have symptoms (Supplementary Figure S4).

To assess disease progression in detached leaves and in wheat ears, we employed a custom-build multispectral phenotyping platform. This platform allows the visualization of diverse physiological traits in real time, based on specific absorption, reflection and emission spectra at a high temporal and spatial resolution at 6 μm. A monochrome camera system including a filter wheel allowed pixel to pixel capturing of Red Green Blue (RGB) values, chlorophyll fluorescence (Chl) and GFP fluorescence (CropReporter, Phenovation, Wageningen, Netherlands). This camera system was mounted on a Cartesian coordinate robot that was housed in a temperature, light and humidity controlled environment. The chamber is equipped with sun LED modules (SLMs) (Phenovation, Wageningen, Netherlands).

Fungal proliferation was assessed based on symptom development using the RGB image. In addition, the effect of disease on the efficiency of photosystem II (F_*V*_/F_*M*_) was assessed (Baker, 2008). Finally, the GFP signal of *F. graminearum* or *F. poae* was used as a hallmark for fungal presence. GFP signals were corrected for auto-fluorescence of senescing leaves and ears resulting in corrected GFP values (cGFP).

Using this approach, images were taken at 3 dai in the screening assay on detached wheat leaves. As the disease progress and symptom development was slower in the ears, images of ears were taken at 4 and 7 dai.

### RNA Extraction and Quantitative Reverse Transcription-PCR

Total RNA from ears was extracted using Trizol reagent (Sigma-Aldrich) according to the manufacturer’s instructions and subsequently quantified with a spectrophotometer (Quantus fluorometer, Promega). First strand cDNA was synthesized using the GoScript^TM^ Reverse Transcription System (Promega). By using GoTaq^®^ qPCR Master Mix (Promega), quantitative reverse transcription (RT-qPCR) analysis was performed using a CFX96 system (Bio-Rad) with the following thermal settings: 95°C for 2 min; 40 cycles of 95°C for 15 s and 60°C for 1 min; melting curve analysis was performed using a temperature profile of heating to 95°C at the rate of 0.5°C per 1 s. Primers used for all genes are listed in Supplementary Table S1. Normalization of defense genes was performed by using the cell division control protein gene (Ta54227) in wheat as reference. Fungal biomass was quantified using a pre-mRNA slicing factor of *F. graminearum* (FGSG_01244) and *F. poae* (FPOA_01282) as described before (Ameye et al., 2015). Gene expression analysis was performed using qBase + software (Biogazelle) based on the fold change which was calculated by dividing the CNRQ values (calibrated normalized relative quantities) of the treated samples by control samples.

### Quantification of Type A and Type B Trichothecenes in Wheat Ears

To investigate whether co-inoculation of *F. poae* and *F. graminearum* on ears impacts the mycotoxin production, ten different mycotoxins produced by either *F. poae* or *F. graminearum* were screened using LC-MS/MS based on (De Boevre et al., 2012).

Briefly, individual mycotoxin solid standards (1 mg) of DON, nivalenol (NIV), 3-acetyl deoxynivalenol (3-ADON), 15-acetyl deoxynivalenol (15-ADON), neosolaniol (NEO), fusarenon-X (FUS-X), T-2 toxin (T-2), HT-2 toxin (HT-2), diacetoxyscirpenol (DAS) and deepoxydeoxynivalenol (DOM) were supplied by Coring System Diagnostics (Gernsheim, Germany) as certified solutions. All mycotoxin solid standards were dissolved in acetonitrile (1 mg mL^–1^), and were storable for a minimum of 1 year at –18°C (Spanjer et al., 2008). Working solutions of 10 ng μL^–1^ for DON, NIV, 3-ADON, 15-ADON, NEO, FUS-X, T-2, HT-2, DAS and DOM were prepared in methanol and stored at –18°C. From the individual working solutions, a mixture was prepared in methanol, stored at –18°C and renewed monthly with the following concentrations: the mycotoxin mix (mycotoxins, 10 ng μL^–1^) and the internal standard (DOM, 10 ng μL^–1^). For the plant tissue, 10 mg of the leaf or 1 g of the ear were crushed using liquid nitrogen. Subsequently, 0.8 mL and 8 mL of the extraction solvent [ethyl acetate/formic acid (99/1, v/v)] were added to each leaf and ear sample, respectively. The mixture was vortexed for 60 s and shaken for 1 h. The samples were centrifuged at 3000 rpm for 15 min, an aliquot of 200 μL from the supernatant was transferred into a glass tube and evaporated under a gentle stream of nitrogen at 40°C till complete dryness. To re-dissolve the residue, 180 μL from the injection solvent (mixture of mobile phase A and B, 60/40, v/v) were added. The tubes were vortexed for 60 s, sonicated for 10 min and the contents were filtered using ultracentrifugation tubes at 10,000 rpm for 5 min. The re-dissolved sample was transferred together with 20 μL of internal standard (DOM, 10 ng μL^–1^) into injection vials, and subjected to LC-MS/MS analysis.

LC-MS/MS analysis was performed using a Waters Acquity UPLC-Quattro Premier XE MS (Waters, Milford, MA, United States) in positive electrospray ionization mode (ESI+) mode. Chromatographic separation was achieved using a Symmetry C18 (150 mm × 2.1 mm, i.d. 5 μm) column with a guard column (10 mm × 2.1 mm i.d., Waters, Zellik, Belgium). The mobile phases consisted of water/methanol/acetic acid (94/5/1, v/v/v) and 5 mM ammonium acetate (mobile phase A), and methanol/water/acetic acid (97/2/1, v/v/v) and 5 mM ammonium acetate (mobile phase B), and were used at a flow rate of 0.3 mL min^–1^ with a gradient elution program. This gradient program was as follow; 0–7 min, 95–35% A; 7–11 min, 35–25% A; 11–13 min, 25–0% A; 13–14 min, 0% A; 14–16 min, 0–40% A; 16–26 min, 40–60% A; 26–28 min, 60–95% A. The column was reconditioned for 5 min before the next injection, with a total analytical run time of 28 min. Data acquisition and processing was facilitated using MassLynx and QuanLynx version^®^ 4.1 software (Micromass, Manchester, United Kingdom). To achieve the optimal selectivity of the MS conditions, data acquisition was performed by applying selected reaction monitoring (SRM). For each target analyte, one precursor and two product ion transitions were selected. The capillary voltage was 3.2 kV and nitrogen was used as the desolvation gas. Source and desolvation temperatures were set at 150 and 350°C, respectively.

### Statistical Analysis

For statistical evaluation and plot generation, the R software version 3.6.0 (RCoreTeam, 2019) and the packages ggplot2 (Wickham, 2016) and agricolae (de Mendiburu, 2014) were used. For multiple comparisons, normality and homoscedasticity assumptions were verified using diagnostic plots. Treatments were statistically compared using ANOVA analyses (with a WELCH correction when homoscedasticity was not met) followed by a *post hoc* Tukey test. All analyses were run at a significance level of *P* = 0.05.

## Results

### *In planta* Screening for Efficient Actinobacterial Strains Against *Fusarium*

**FIGURE 1:**
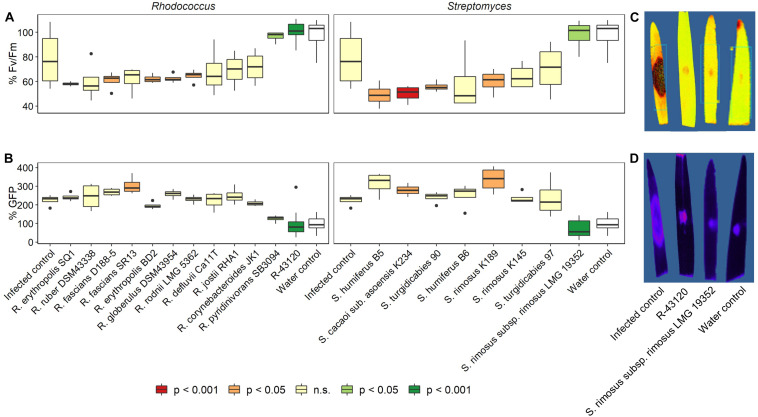
Biocontrol capacity of *Streptomyces* and *Rhodococcus* strains against *F. graminearum* as the main causal agent of FHB. Strains were co-applied with GFP tagged *F. graminearum* PH-1 in a detached leaf assay. **(A)** % Fv/Fm (percentage increase/decrease in efficiency of photosystem II) compared to mock-inoculated control plants; values represent symptom development based on the efficiency of photosystem II. Strains with significant positive biocontrol capacities are indicated in green. **(B)** Assessment of the relative active fungal biomass based on the GFP signal of the mock infected control plants. **(C)** Picture of R-43120 and LMG 19352 treatment based on the efficiency of photosystem II. **(D)** Picture of R-43120 and LMG 19352 treatment based on the GFP signal. Significant differences (positive or negative, based on the *P*-values) are indicated by the colors of the boxes. Boxplots indicate the median (horizontal lines), 25th and 75th percentile range (boxes) and up to 1.5 × IQR (Interquartile Range) (whiskers).

Using an in house developed detached leaf assay (Ameye et al., 2015), the *in planta* biocontrol capacity of several actinobacterial strains comprising both *Rhodococcus* sp. and *Streptomyces* sp. was assessed against the main causal FHB pathogen *F. graminearum*. The strains were first identified using MALDI T-MS (data not shown). Results of the detached leaf assay are shown in Figure 1. A wide variety of interactions was observed ranging from a complete blocking of infection to enhanced disease development. Symptom development (Fv/Fm, chlorophyll fluorescence) was completely blocked for *Rhodococcus* sp. R-43120 and *Streptomyces rimosus* LMG 19352 (Figures 1A,C). In addition, the GFP signal was absent and resembled the mock inoculated control treatment (Figures 1B,D). With some bacterial strains, a more severe infection was observed than in the uninfected control treatment. This was the case for *R. fascians* D188, *R. erythropolis* BD2, *R. globerolus* DSM 43954, *R. rodnii* LMG 5362, *S. humiferus* B5, *S. cacaoi* subsp. a*soensis* K234, *S. turgudicabies* 90, and *S. rimosus* K189. Some of the actinobacterial strains resulted in an increased cGFP signal of *F. graminearum* (*R. fascians* SR13; *S. cacaoi* subsp. *asoensis* K234; *S. rimosus* K189). This points to a proliferated growth of *F. graminearum* due to the presence of these actinobacterial strains.

Based on these results, two actinobacterial strains *Streptomyces rimosus* LMG 19352 and *Rhodococcus* sp. R-43120 were further used in this paper to investigate their modes of action, their interaction with the two main members of the FHB complex *F. graminearum* and *F. poae in vitro* and *in planta*.

### *In vitro* Antagonism of *Streptomyces rimosus* LMG 19352 (b1) and *Rhodococcus* sp. R-43120 (b2)

**FIGURE 2:**
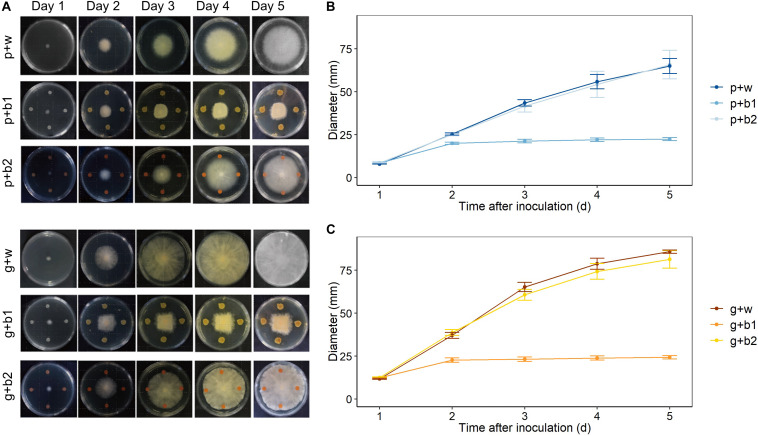
Effect of *Streptomyces rimosus* strain LMG 19352 (b1) and *Rhodococcus sp*. R-43120 (b2) on the growth of *F. graminearum* PH-1 and *F. poae* 2516 on TSA plates. Treatments: p + w (Inoculation with *F. poae* and water); p + b1 (Inoculation with *F. poae* and LMG 19352); p + b2 (Inoculation with *F. poae* and R-43120); g + w (Inoculation with *F. graminearum* and water); g + b1 (Inoculation with *F. graminearum* and LMG 19352); g + b2 (Inoculation with *F. graminearum* and R-43120). **(A)** Images taken from day 1 till day 5. **(B)** *F. poae* growth from day 1 till day 5, *n* = 5 (five biological reps per time point). **(C)** *F. graminearum* growth from day 1 till day 5, *n* = 5 (five biological reps per time point).

The direct antagonistic effect of *Streptomyces rimosus* strain LMG 19352 (b1) and *Rhodococcus* sp. strain R-43120 (b2) was assessed against *F. poae* 2516 and *F. graminearum* PH-1 in co-cultivation assays on TSA medium. These analyses revealed that biocontrol strain b1 displayed a very strong antagonistic activity against *F. graminearum* PH-1 (g + b1) as well as against *F. poae* 2516 (p + b1) (Figure 2) and against a combined inoculum of *F. poae* and *F. graminearum* (pg + b1) (Supplementary Figure S1). The antagonistic effects were visible from day 2 onward and were identical against both *F. poae* and *F. graminearum*. For biocontrol strain b2, there was no effect on the growth of *F. poae* (p + b2) or *F. graminearum* (g + b2) (Figure 2). Similar results were obtained in the co-inoculations of *F. poae* and *F. graminearum* (Supplementary Figure S1). The *in vitro* inhibition of *F. poae* and *F. graminearum* by b1 was statistically evaluated by comparing the slope of the growth curves in the linear region (day 2–4 after inoculation). In the presence of the b1 antagonist, fungal growth of *Fusarium poae, Fusarium graminearum* and combined *F. poae* and *F. graminearum* were significantly reduced, resulting in significant lower slopes of the linear regression curves (ANOVA, all *p*-values < 0.001). Strain b2 showed no significant effect compared to the water control (*p* = 0.980, *p* = 0.218, and *p* = 0.184 resp.).

### Impact of *Streptomyces rimosus* LMG 19352 (b1) and *Rhodococcus* sp. R-43120 (b2) on FHB on Wheat Ears Infected With *F. graminearum*, *F. poae* or a Combined Infection of *F. graminearum* and *F. poae*

**FIGURE 3:**
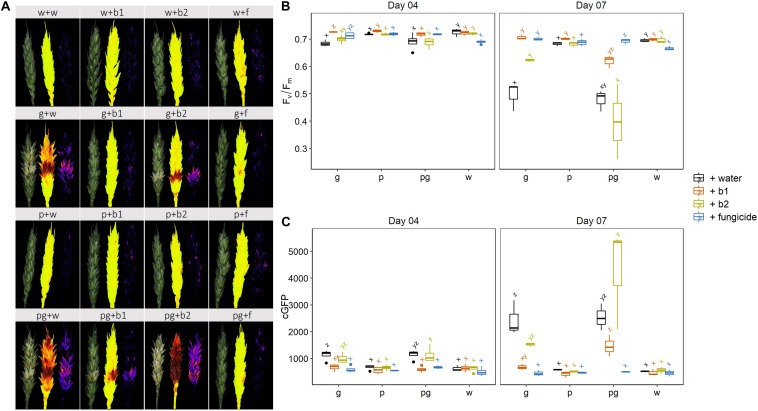
**(A)** Visual symptoms on wheat ears after infection by *F. graminearum* PH-1 (g), *F. poae* 2516 (p) or a combination of both pathogens (pg) and treated with water (+w), with *S. rimosus* LMG 19352 (+b1), with *Rhodococcus sp*. R-43120 (+b2) or with the fungicides prothioconazole + spiroxamine (f) at 7 dai. **(B)** Results of quantitation of the Fv/Fm (efficiency of photosystem II) signal at 4 and 7 dai, *n* = 6 (six biological reps per treatment and per pathogen combination). **(C)** Results of quantitation of the cGFP (corrected GFP value) signal (background signal) at 4 and 7 dai, *n* = 6 (six biological reps per treatment and per pathogen combination). Boxplots indicate the median (horizontal lines), 25th and 75th percentile range (boxes) and up to 1.5 × IQR (Interquartile Range) (whiskers). Different letters indicate significant differences (*P* < 0.05) between treatments for each time point within the same pathogen combination. The experiment was repeated twice in time.

We assessed the impact of biocontrol strains b1 and b2 when applied with *F. graminearum* (g + b1; g + b2), *F. poae* (p + b1; p + b2), or a combination of both FHB members (pg + b1; pg + b2). The fungicide prothioconazole + spiroxamine (g + f; p + f; pg + f) was included as a control treatment. The disease was assessed by multispectral analysis of the dark-adapted Fv/Fm chlorophyll fluorescence or by the GFP signal. Results are shown in Figure 3.

At 4 dai, a slight but significant phytotoxic effect was observed in the mock inoculated ears treated with the fungicide which was hallmarked by a reduction in Fv/Fm. This slight phytotoxic effects were not observed in the combined treatments with both pathogens. In the *F. graminearum* infected ears, a small but significant biocontrol effect was observed for biocontrol strain b1 and the fungicide prothioconazole + spiroxamine, whereas b2 showed Fv/Fm values comparable to the control (Figure 3B). Finally, no biocontrol effects by either of both strains were observed in the ears co-inoculated with *F. graminearum* and *F. poae.* In parallel with symptoms (Fv/Fm) the fungal presence was also assessed through its GFP signal. Results of this analysis are shown in Figure 3C. The results of the *F. graminearum* biomass assessment through its GFP signal were similar to the Fv/Fm results.

At 7 dai, the mock-inoculated controls confirmed the very mild but significant negative effects of the fungicide treatment on the Fv/Fm values. In addition, it was clear that a single infection of wheat ears with *F. graminearum*, was efficiently controlled by both b1, b2 and the fungicide treatment which resulted in Fv/Fm values comparable to the mock inoculated control ears. Interestingly, the GFP signal indicated that b2 showed intermediate effects on the *F. graminearum* biomass, with values between the mock inoculated and inoculated control treatments. In contrast, the *F. graminearum* biomass in combination with b1 was strongly reduced and comparable to the fungicide treatment. In all the singular *F. poae* inoculations, no symptoms developed and Fv/Fm values of ears were not significantly different from the mock inoculated ears (Figures 3A,B).

Remarkably, despite the fact that the infection levels between the singular *F. graminearum* inoculation and the combined infection of *F. graminearum* and *F. poae* were not statistically different at Fv/Fm level or GFP level (*p*-values were respectively *p* = 0.123 and *p* = 0.405 after an independent sample *t*-test), none of the two biocontrol agents b1 or b2 displayed the same biocontrol capacity as observed in the singular *F. graminearum* infection. Lower Fv/Fm values were observed in the b1 and b2 treatments after dual infection with *F. poae* and *F. graminearum* compared to a singular infection with *F. graminearum* with *p*-values of 0.031 and 0.024 respectively (independent sample *t*-test). In addition, the Fv/Fm values of ears co-inoculated with *F. poae* and *F. graminearum* and treated with b1 and b2 were not significantly different from the infected controls. When assessing the GFP signal, an even more interesting phenomenon was observed for b1 and b2. In the combined infections of *F. poae* and *F. graminearum*, this biocontrol agent resulted in a consistent, significant increase in GFP signal compared to the singular *F. graminearum* infected control ears (with one-sided *p*-values of 0.015 and 0.040 respectively, *t*-test). This result shows that the efficacy of the biocontrol strains in regard to symptom development and fungal presence assessed through the GFP tag, is reduced when the FHB disease complex comprises both *F. poae* and *F. graminearum* (Figure 3).

Subsequently, we assessed the actively growing *F. poae* and *F. graminearum* through RT-qPCR of household genes (Supplementary Figure S2). These data show that biocontrol strain b1 efficiently reduces actively growing *F. poae* and *F. graminearum* on wheat ears. In addition, b2 does have an intermediate effect on *F. poae* when comparing with the *F. poae* infected water control, while no effect on *F. graminearum* PH-1 was observed. Again, similar as for Fv/Fm and GFP, the biocontrol strain b2 was less effective in reducing the biomass in the combined infection of *F. poae* and *F. graminearum* compared to a singular *F. graminearum* infection (*p*-value = 0.034, one-sided independent sample *t*-test).

In conclusion, the impact of the fungicide treatment seems independent of the FHB disease complex constitution, while both biocontrol treatments seemed less effective on *F. graminearum* when the latter was co-applied with the weak pathogen *F. poae*.

### Impact of *Streptomyces rimosus* LMG 19352 (b1) and *Rhodococcus* sp. R-43120 (b2) on Type A and Type B Trichothecene Production When Co-applied With *Fusarium* sp. on Wheat Ears

**FIGURE 4:**
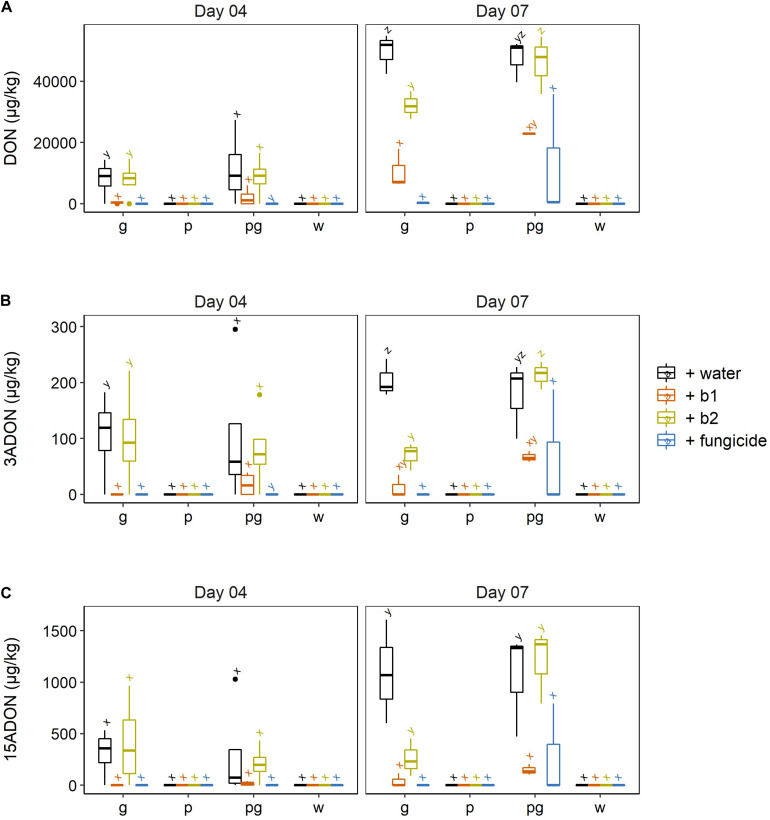
**(A)** DON, **(B)** 3ADON, and **(C)** 15ADON concentrations in wheat ears after infection by *F. graminearum* PH-1 (g), *F. poae* 2516 (p) or a combination of both pathogens (pg) and treated with water (+w), with *S. rimosus* LMG 19352 (+b1), with *Rhodococcus sp*. R-43120 (+b2) or with the fungicides prothioconazole + spiroxamine (f). Boxplots indicate the median (horizontal lines), 25th and 75th percentile range (boxes) and up to 1.5 × IQR (Interquartile Range) (whiskers). Different letters indicate significant differences (*P* < 0.05) between treatments for each time point within the same pathogen combination. The experiment was repeated twice in time.

We studied the impact of the biocontrol strains and the fungicide treatment on mycotoxin production by both *F. poae* and *F. graminearum.* None of the type A or type B trichothecenes known to be produced by *F. poae* 2516 were detected in any of the treatments. So in none of the interactions nivalenol (NIV); diacetoxyscirpenol (DAS) or neosolaniol (NEO) were observed above the detection limit. For the *F. graminearum* mycotoxins, we assessed the presence of the type B trichothecenes DON, 3-ADON and 15-ADON (Figure 4).

At 4 dai, the ears that were infected by a singular *F. graminearum* infection and treated with the fungicide or the biocontrol agent b1 showed a significant decrease in DON. In contrast application of biocontrol strain b2 resulted in DON levels not significantly different from the inoculated singular *F. graminearum* control ears. Similar observations were done in regard to 3-ADON and 15-ADON although the differences of the latter ones were not significant.

In the dual inoculation of *F. graminearum* and *F. poae*, no significant reduction of DON nor its derivatives were observed due to the presence of biocontrol agents b1 and b2. Nevertheless, a non-significant reduction was observed for DON, 3-ADON, and 15-ADON for b1.

This trend was also apparent at 7 dai. In the singular *F. graminearum* infections, the application of b1 resulted in a significant reduction of DON, which was comparable to the reduction by the fungicide treatment. The biocontrol strain b2 resulted in a significant reduction of DON, but this reduction was significantly less pronounced than the reduction realized by b1. Similar conclusions could be drawn for 3-ADON (Figure 4B) and 15-ADON (Figure 4C).

At 7 dai, the impact of both biocontrol strains b1 and b2 on the dual inoculations of *F. graminearum* and *F. poae* was different compared to their effect in the singular *F. graminearum* interaction. Firstly, the capacity of b2 to reduce DON, 3-ADON, and 15-ADON was completely lost and the levels of these mycotoxins were not significantly different from levels in the infected control ears. In addition, also b1’s capacity to reduce *F. graminearum*, DON and its acetylated derivatives in the co-inoculations of *F. graminearum* and *F. poae* were significantly reduced compared to the singular *F. graminearum* inoculation (two-sided *p*-values = 0.029 and 0.013 for DON and 3-ADON, a one-sided *p*-value of 0.045 for 15-ADON).

Also the impact of the prothioconazole + spiroxamine fungicide treatment on DON, 3-ADON and 15-ADON levels appeared different between the singular *F. graminearum* versus dual infection with *F. graminearum* and *F. poae.* In the singular *F. graminearum* infection hardly any toxin was detected while in the dual infection with *F. graminearum* and *F. poae* sometimes high amounts were observed for all three toxins although this difference was not significant due to the high variability in the toxin data in the latter interaction (Figure 4). These data on the chemical control of FHB demonstrate that the efficacy of the chemical control shows an increased variability and as such is less consistent when both *F. poae* and *F. graminearum* are present in the FHB disease complex.

### Impact of *Streptomyces rimosus* LMG 19352 (b1) and *Rhodococcus* sp. R-43120 (b2) on Plant Derived DON-3G When Co-applied With *Fusarium* spp. on Wheat Ears

**FIGURE 5:**
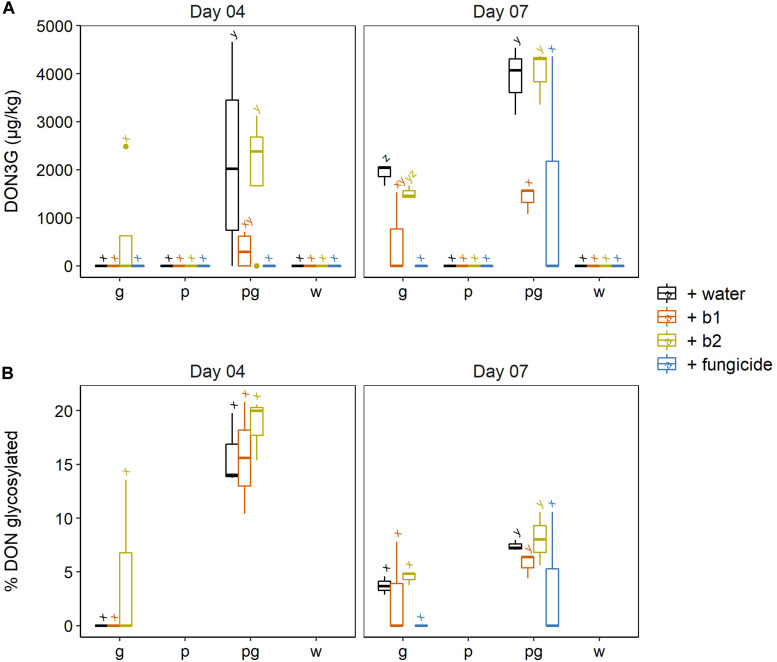
**(A)** DON-3G and **(B)** percentage of DON being glycosylated in wheat ears after infection by *F. graminearum* PH-1 (g), *F. poae* 2516 (p) or a combination of both pathogens (pg) and treated with water (+ w), with *S. rimosus* LMG 19352 (+b1), with *Rhodococcus sp*. R-43120 (+b2) or with the fungicides prothioconazole + spiroxamine (f). Boxplots indicate the median (horizontal lines), 25th and 75th percentile range (boxes) and up to 1.5 × IQR (Interquartile Range) (whiskers). Different letters indicate significant differences (*P* < 0.05) between treatments for each time point within the same pathogen combination. The experiment was repeated twice in time.

As DON is a phytotoxin, plants have developed detoxification strategies for DON (Berthiller et al., 2013). One of the best described strategies for plants to cope with DON is the glucosylation of DON, after which DON-3-glucoside (DON3G) is directed to the vacuole (Audenaert et al., 2014). In this respect, we also checked whether the presence of biocontrol strains affected the plant’s capacity to glucosylate DON (Figure 5A). Here, the results were less clear: although b1 resulted in a lower ratio of glucosylated DON, there were no significant differences between treatments (Figure 5B).

At 4 dai, the singular *F. graminearum* inoculations did not result in any accumulation of DON-3G, with the exception of 1 positive sample in the ears treated with b2. On the contrary, in the dual inoculations with *F. graminearum* and *F. poae*, all treatments resulted in the accumulation of DON-3G and the highest DON-3G levels were observed in the water- and b2 treated wheat ears (Figure 5A). This difference in glucosylation was not due the fact that less DON, 3-ADON or 15-ADON had been formed in the singular *F. graminearum* interactions as the content of these trichothecenes was the same in the singular *F. graminearum* inoculations versus the dual *F. graminearum* and *F. poae* inoculations. This points to a higher DON glucosylation rate in the dual inoculations independently of the water, b1, b2 or fungicide treatment.

Also at 7 dai, both DON-3G levels and DON glucosylation ratios per treatment (water, b1, b2 or fungicide) were always higher in the dual inoculation of *F. poae* and *F. graminearum* compared to the singular *F. graminearum* infection (Figures 5A,B). These differences in DON-3G levels were not attributed to the fact that less DON, 3-ADON or 15-ADON had been formed in the singular *F. graminearum* interactions, as the content of these trichothecenes was the same in the singular *F. graminearum* inoculations versus the dual *F. graminearum* and *F. poae* inoculations (*cf*. Figure 4). This points to a higher DON glucosylation rate in the dual inoculations, independently of the water, b1, b2 or fungicide treatment. At 7 dai, the increased ratio of DON-3G in ears co-inoculated with *F. graminearum* and *F. poae*, compared to ears inoculated with *F. graminearum* alone, was observed for all treatments (water, b1, b2, fungicide) although it was not significant for the fungicide treatment due to the high variability of the data.

Wheat ears treated with biocontrol strain b1 resulted in almost no symptom development. Counterintuitively, this strain showed the lowest DON-3G ratios in wheat ears which shows that DON-glucosylation is probably not involved in symptom reduction by *S. rimosus* LMG19352.

### Impact of *Streptomyces rimosus* LMG 19352 (b1) and *Rhodococcus* sp. R-43120 (b2) on Key Genes in the Biosynthesis of Jasmonic Acid and Salicylic Acid

Timely upregulation of *ICS*- and *LOX* genes can result in a good control of FHB symptoms caused by *F. graminearum* (Ameye et al., 2015) and *F. poae* interferes with these processes and thereby influences symptom development by *F. graminearum* (Tan et al., 2020). Hence we wanted to assess the involvement of these genes in the biocontrol of b1 and b2 and in the chemical control by the fungicide application. Therefore, the expression levels were determined of genes encoding hallmark enzymes phenylalanine ammonia-lyase (PAL) and isochorismate synthase (*ICS*) (for the SA biosynthesis) and lipoxygenases *LOX1* and *LOX2* (for the biosynthetic pathway leading to JA).

**FIGURE 6:**
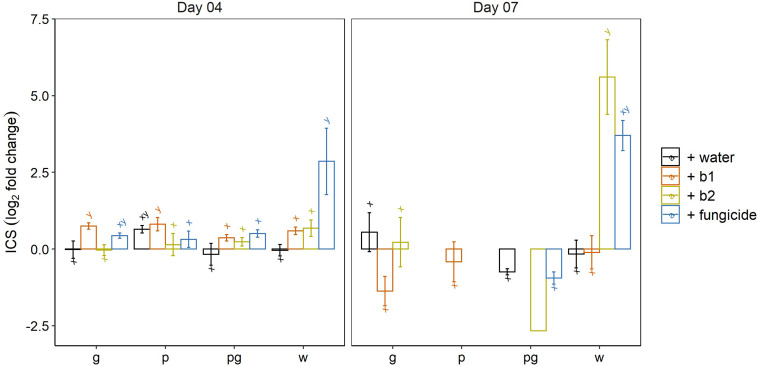
Expression profile of isochorismate synthase (ICS) at 4 and 7 dai by *F. graminearum* PH-1 (g), *F. poae* 2516 (p) or a combination of both pathogens (pg) and treated with water (+w), with *S. rimosus* LMG 19352 (+b1), with *Rhodococcus sp*. R-43120 (+b2) or with the fungicides prothioconazole + spiroxamine (f). Bars represent log_2_-transformed means of four biological replicates; error bars indicate ± 1 standard error. Different letters indicate significant differences (*P* < 0.05) between treatments for each time point within the same pathogen combination. Fold change was calculated by dividing the CNRQ values (calibrated normalized relative quantities) of the treated ears by values of the control ears. Normalization of defense genes was performed by using the cell division control protein gene (Ta54227) in wheat as reference. Missing bars for the *F. graminearum* (g), the *F. poae* (p) and the combined infection (pg) point to an ICS expression level (and Ct value) below the limit of detection. The experiment was repeated twice in time.

At 4 dai, we observed a significant induction of *ICS* by the fungicide (prothioconazole + spiroxamine) compared to the mock inoculated water treatment (Figure 6). In addition, a small but significant upregulation of *ICS* was observed in the singular *F. graminearum* infected wheat ears treated with biocontrol strain b1. Finally, when considering the impact of the pathogen alone on the expression of *ICS*, a small but significant upregulation of *ICS* compared to mock inoculated wheat ears was observed after singular inoculation of wheat ears with *F. poae* but not of *F. graminearum* in the water treatment (*p* = 0.014 and *p* = 0.993, respectively independent sample *t*-test).

At 7 dai we observed a significant upregulation of *ICS* in the mock inoculated wheat ears treated with biocontrol strain b2. This induction of *ICS* was comparable to the induction of *ICS* by the fungicide prothioconazole + spiroxamine. However, the latter one was not significantly different from the control due to variability in the data. This result shows that biocontrol strain b2 triggers the SA biosynthesis pathway. Remarkably, the induction of *ICS* disappeared in all interactions of b2 in which a pathogen was involved (*F. graminearum* and/or *F. poae*). Similar expression analyses were done for phenylalanine ammonia lyase (*PAL*). At 4 dai, an induction of *PAL* was observed in all treatments comprising a singular *F. graminearum* inoculation. At 7 dai, only the combined application of b2 and *F. graminearum* remained significant when comparing with the mock-inoculated control treatment (Supplementary Figure S3).

**FIGURE 7:**
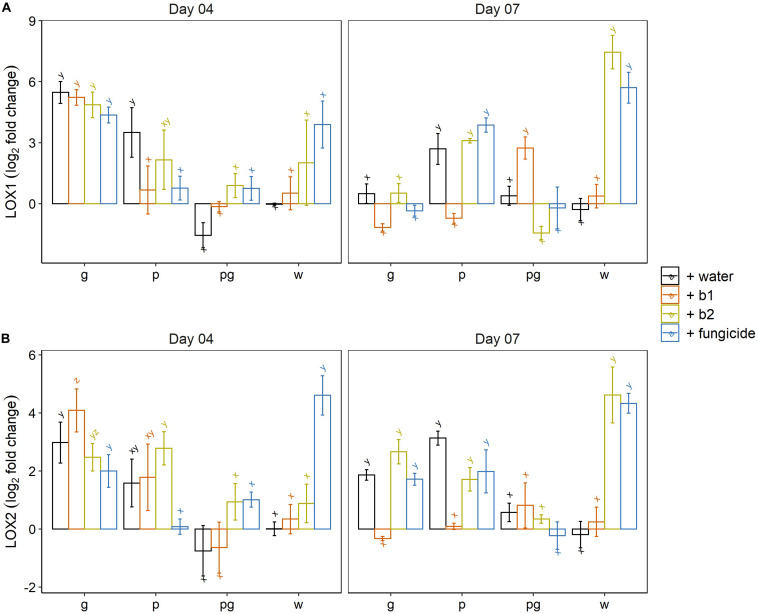
Expression profile of Lipoxygenase 1 **(A)** and Lipoxygenase 2 **(B)** (LOX1 and LOX2) at 4 and 7 dai by *F. graminearum* PH-1 (g), *F. poae* 2516 (p) or a combination of both pathogens (pg) and treated with water (+w), with *S. rimosus* LMG 19352 (+b1), with *Rhodococcus* sp. R-43120 (+b2) or with the fungicides prothioconazole + spiroxamine (f). Bars represent log_2_-transformed means of four biological replicates; error bars indicate ± 1 standard error. Different letters indicate significant differences (*P* < 0.05) between treatments for each time point within the same pathogen combination. Fold change was calculated by dividing the CNRQ values (calibrated normalized relative quantities) of the treated ears to the control ears. Normalization of defense genes was performed by using the cell division control protein gene (Ta54227) in wheat as reference. The experiment was repeated twice in time.

Jasmonic acid mediated plant defense was monitored by following the expression profiles of lipoxygenases 1 and 2. At 4 dai, a clear induction of *LOX1* was observed in the mock-inoculated wheat ears treated with the fungicide prothioconazole + spiroxamine and wheat ears treated with the biocontrol strain b2. Both inductions were highly variable and therefore not significantly different from the mock-inoculated water control (Figure 7A). We attribute this high variability to the fact that 4 dai is a critical time point in which in some ears *LOX1* gene expression has been triggered while in others, the level of *LOX1* expression is still at basal levels. A clear and significant induction of *LOX1* was observed in all treatments of the singular *F. graminearum* inoculated wheat ears and in the water and b2 treated ears inoculated with *F. poae* which confirmed our previous results on the induction of *LOX1* by *F. poae* and *F. graminearum* (Tan et al., 2020). In addition, per treatment (water, b1, b2 and fungicide) this induction of *LOX1* by singular inoculations by *F. graminearum* was suppressed when *F. graminearum* was co-inoculated with *F. poae* (Figure 7A).

At 7 dai, a clear and consistent induction of *LOX1* was observed in the mock-inoculated controls treated with b2 and the fungicide prothioconazole + spiroxamine. The wheat ears that were inoculated by *F. poae* all showed an induction of *LOX1* with the exception of the b1 treatment. The biocontrol agent b1 resulted in a slight repression of *LOX1* gene expression compared to the mock-inoculated water control.

For *LOX2* at 4 dai, the fungicide prothioconazole + spiroxamine resulted in a significant upregulation of *LOX2* compared to the mock inoculated water control. In addition, the induction of *LOX2* by a singular *F. graminearum* and *F. poae* infection was observed. Within the singular *F. poae* interactions, the induction of *LOX2* in all treatments except for the fungicide treatment was significant. Again, the induction of *LOX2* that was observed in the singular inoculations, was suppressed in all treatments in which *F. graminearum* was co-inoculated with *F. poae*.

At 7 dai, a significant induction of *LOX2* was observed in the mock-inoculated control ears treated with the biocontrol strain b2 or the fungicide. In the singular *F. poae* and *F. graminearum* inoculations, the induction of *LOX2* was observed in the water, b2 and fungicide treatments while the application of biocontrol strain b1 resulted in a slight suppression of *LOX2* gene expression. In all treatments (water, b2 and fungicide) the induction of *LOX2* that was observed in the singular inoculations, was suppressed when *F. graminearum* was co-inoculated with *F. poae.*

In conclusion, the ICS, *LOX1* and *LOX2* expression data show that biocontrol strain b2 and the fungicide prothioconazole + spiroxamine induce ICS, *LOX1* and *LOX2* even in absence of a pathogen. Secondly, a singular *F. graminearum* inoculation resulted in an induction of *LOX1* and *LOX2* gene expression independently of the treatment (water, b1, b2 and fungicide). Similar results were obtained for the singular *F. poae* inoculations although the b1 treatment for *LOX1* and the fungicide treatment for *LOX2* did not show this induction. Finally, co-inoculation of *F. graminearum* and *F. poae* resulted in *LOX1* and *LOX2* expression levels similar to the expression levels in mock-inoculated control ears which points to a complete suppression of JA-dependent defense in wheat ears inoculated with *F. poae* and *F. graminearum.*

## Discussion

When a plant is infected by a pathogen, a complex interaction between plant, pathogen and the residing microbial community starts (e.g., del Barrio-Duque et al., 2020). The outcome of this interaction is then typically determined by the mutual interplay of plant, microbial and plant pathogen-derived signals and pathways. The knowledge that plant pathogens can be controlled through the action of other microbes has spurred researchers to explore the use of these antagonistic bacteria and fungi as biocontrol agents against fungal, bacterial, and oomycete pathogens. Their modes of action comprise direct antagonism, antibiosis, mycoparasitism, competition for niche, and induction of the plant’s intrinsic plant defense amongst others (De Silva et al., 2019; Khan et al., 2020; Raymaekers et al., 2020).

Yet, to date, no in-depth information is available on the impact of chemical control agents or biocontrol agents on the outcome of plant diseases caused by complexes of microbes from the same or other genus (Lamichhane and Venturi, 2015). Nevertheless, such disease complexes comprise devastating diseases such as Black sigatoka disease in banana caused by several *Mycosphaerella* sp. (Arzanlou et al., 2007), potato leaf spot which is caused by a complex of *Alternaria* sp. (Vandecasteele et al., 2018), rice sheath rot disease caused by *Fusarium* sp. and *Sarocladium oryzae* (Bigirimana et al., 2015) and the FHB disease in wheat which is considered in the present paper. Studies on control strategies to control these fungal disease complexes are still at their infancy and to our knowledge when available, studies on biocontrol strategies only target one member of a disease complex rather than looking at the impact of biocontrol strains on the multiple members of such a disease complex. For FHB, focus is commonly on *F. graminearum* and biocontrol agents against this pathogen have been described in detail. *Streptomyces* sp. (Palazzini et al., 2017), *Pseudomonas* sp. (Wang et al., 2015; Chen et al., 2018), *Bacillus* sp. (Palazzini et al., 2007), and fungal strains *Trichoderma* sp. (Mahmoud, 2016; Sarrocco et al., 2020), *Cryptococcus* sp. (Zhang et al., 2007), and *Clonostachys* sp. (Xue et al., 2014; Demissie et al., 2020) are examples of a non-exhaustive list of biocontrol agents that have been shown to reduce FHB symptoms by *F. graminearum*. However, the impact of other members residing in the FHB disease complex on the biocontrol efficacy of biocontrol strains was not studied so far.

In present work, we used Actinobacteria belonging to the genera of *Streptomyces* and *Rhodococcus* to investigate their impact on FHB caused by a complex of *F. poae* and *F. graminearum.* A fungicide with prothioconazole + spiroxamine as active ingredients was included as a control. In wheat ears, both strains were good biocontrol agents against a singular *F. graminearum* infection although b1 showed a better control capacity based on the reduction of the fungal biomass than b2. However, when applied in wheat ears co-inoculated with *F. poae* and *F. graminearum*, both biocontrol strains had significantly reduced biocontrol capacity, which shows that the presence of *F. poae* hampers the biocontrol of *F. graminearum* by both strains. This finding was striking as both biocontrol strains had different modes of action and interacted differently with the wheat plant. Moreover, *Streptomyces rimosus* LMG 19352 (b1) showed a clear antagonism with *F. graminearum* (Figure 2) and *Rhodococcus* sp. R-43120 (b2) showed a significantly induction of plant defense (Figures 6, 7).

The reduced biocontrol efficacy was also observed at the level of the mycotoxin production by *F. graminearum* in the combined inoculations with *F. poae*. In search for an explanation, we found that this reduced control of *F. graminearum* was not mediated by *F. poae* producing type A and type B trichothecenes (NIV, DAS, NEO) during its colonization as these trichothecenes were not present at detectable levels in wheat ears. For the chemical control using prothioconazole + spiroxamine, a decreased efficacy was observed for accumulation of DON, 15-ADON and 3-ADON in ears colonized by a combination of *F. graminearum* and *F. poae* compared to the singular *F. graminearum* infections. This result shows that efficacy of chemical control in FHB depends on the complexity of the disease complex and that some Fusarium species might hamper a good control.

When assessing the plant defense response, we observed that *Rhodococcus* sp. R-43120 (b2) was shown to induce *ICS*, *LOX1*, and *LOX2* genes in mock inoculated wheat ears which shows that this strain can trigger the innate plant’s defense system especially at later timepoints after application. The induction of *LOX* genes by Actinobacteria is not new. Previously, Abbasi et al. (2019) demonstrated an activation of plant defense including the induction of *LOX* genes by Actinobacteria. In order to link this observation with the biocontrol capacity of the strain, we investigated the expression of the *LOX* genes in the presence of both *Fusarium* pathogens. When looking at the expression levels of *LOX1* and *LOX2* at 4 dai, both genes were induced by *F. poae* and *F. graminearum* independently of the presence of biocontrol strain *Rhodococcus* sp. R-43120 (b2) while the combined *F. graminearum* and *F. poae* inoculations in absence or presence of b2 resulted in the induction of *LOX1* or *LOX2*. LOX1 lacks a chloroplast targeting peptide while LOX2 is targeted to the chloroplast (Feng et al., 2010). At 7 dai, the expression got more differential and we observed that b2 induced both *LOX1* and *LOX2* in the mock inoculated controls and in the singular *F. poae* infections. In addition, the *F. poae* inoculation itself results in the induction of both *LOX* genes. However, in the interaction with *F. graminearum* only *LOX2* was induced, while *LOX1* was not significantly different from the mock inoculated controls. Finally, in the interaction with the dual infection with both *F. poae* and *F. graminearum* neither of the *LOX* genes was significantly induced. As in the latter interaction, no biocontrol effect of b2 was observed while the strain showed good biocontrol effects in the singular *F. graminearum* interaction, we hypothesize that especially the induction of *LOX2* could be crucial for the biocontrol effect of biocontrol strain b2, while *LOX1* is probably not involved in the biocontrol trait. The role of 9-LOXs in susceptibility of wheat and in DON production by *F. graminearum* has previously been established (Nobili et al., 2014; Nalam et al., 2015). In the past, 13-LOXs have been associated with high basal resistance of the well-known wheat cultivar Sumai 3 (Zhuang et al., 2013). From work in other crops, it is known that 9-LOXs are targets for microbial effectors which can lead to transcriptional reprogramming and subsequent increased resistance or susceptibility (Battilani et al., 2018). In addition, in maize it has been shown that 9-LOXs and 13-LOXs play reciprocal roles in the susceptibility or resistance against respectively, *F. verticillioides* and *A. flavus* (Gao et al., 2009). We want to pinpoint that it is known that LOXs are found to yield mixtures of 9- or 13 hydroperoxide variants but that the presence of a chloroplast targeting peptide in LOX2 versus LOX1 is discriminative (Feng et al., 2010).

The biocontrol of *F. graminearum* by application of b2 through induction of *LOX* genes and *ICS* confirms the importance of SA-and JA-dependent defense responses against this hemi-biotrophic pathogen. It has been shown previously that a meticulously time-dependent up-and down regulation of SA- and JA-dependent defenses determines the outcome of *F. graminearum* in wheat (Ameye et al., 2015).

In addition, we have previously demonstrated that *F. poae*, when present in the FHB disease complex together with *F. graminearum*, influences the outcome of the *F. graminearum* infection by modulating the SA- and JA derived plant defenses (Tan et al., 2020). Small distortions in these interconnected pathways might have serious impacts on the outcome of the interaction of wheat with *F. graminearum*. These small aberrancies can be different environments, control strategies, different timing of infection of FHB members or different composition of the FHB disease complex. Several of these components have been nicely reviewed by Xu and Nicholson (2009).

In addition, these aberrations can comprise cross kingdom interactions. This was also reported in a different study, where we investigated an inter-kingdom interaction between wheat, *F. graminearum* and *Sitobion avenae* (English grain aphid) (De Zutter et al., 2017). In that work, pre-infestation of wheat ears with aphids resulted in a down regulation of PAL at early time points, which resulted in a suppression of PAL during a subsequent *F. graminearum* infection and a more proliferated outgrowth of the fungal pathogen compared to a single *F. graminearum* infection (De Zutter et al., 2017).

For the chemical control, we could demonstrate that in mock-inoculated wheat ears that were treated with prothioconazole + spiroxamine a clear induction of *LOX1, LOX2*, and *ICS* was observed. Knowledge on the impact of fungicides at the level of the intrinsic plant defense system is still scarce and unexplored. Our results suggest that the chemical control of *F. graminearum* might be the result of a combination of fungicidal effects and plant-mediated antifungal responses.

Because especially biocontrol strain b2 interfered with the plant defense mechanism at the level of *LOX-* and *ICS* gene expression, we further explored the presence of DON-3G, a metabolite resulting from the glucosylation of DON by the plant enzyme uridine diphosphate-glucosyltransferase. From literature, it is known that glucosylation in wheat is associated with the quantitative trait locus Fhb1. Moreover, wheat cultivars carrying this locus have higher DON-3G/DON ratios (Berthiller et al., 2013; Audenaert et al., 2014; Kluger et al., 2015). Finally, it is known that several biocontrol agents such as *Clonostachys rosea* can increase the plant’s capacity to glucosylate DON (Abdallah et al., 2018). Despite the fact that DON glucosylation is frequently reported in literature as an efficient strategy of plants or biocontrol agents to detoxify DON, no clear induction of DON glucosylation was observed in wheat ears upon application of biocontrol strains b1, b2 or the fungicide prothioconazole + spiroxamine in our experiments. However, co-inoculation of wheat ears by *F. poae* and *F. graminearum* resulted in an increased DON-3G/DON ratio compared to singular *F. graminearum* inoculations. This observation was found for all treatments (water, b1, b2 and fungicide indicating that the presence of *F. poae* in the disease complex results in an increased DON-glucosylation activity.

In conclusion, we have shown that *Streptomyces rimosus* LMG 19352 and *Rhodococcus* sp. R-43120 efficiently reduced *F. graminearum* symptoms in wheat ears. In addition, we demonstrated that the biocontrol capacity of these actinobacterial strains and to a lesser extent the fungicide prothioconazole + spiroxamine was critically hampered by the presence of the weakly pathogenic *F. poae*. As a more generic conclusion, our study shows that integrated control strategies against FHB and other plant diseases that are caused by a multiple fungi might be far more complex and difficult than initially thought due to the presence of multiple species in these so-called pathobiomes, calling for an articulation of Koch’s postulates. As we start to unravel the complex aspects of pathobiomes, it may become clear how these species complexes operate in a multiplayer mode to cause symptoms in plants.

## Data Availability Statement

The original contributions presented in the study are included in the article/Supplementary Materials, further inquiries can be directed to the corresponding author/s.

## Author Contributions

JT, MA, and KA designed the experiments. JT performed all the experiments. ND performed the image and data analysis. PV and AW coordinated the identification of the actinobacterial isolates. SDS, TT, and MD interpreted the mycotoxin data. JT, MA, KA, CW, TVDL, and MD wrote the manuscript. ND, CW, TVDL, and AW read and revised the manuscript. All the authors contributed to the article and approved the submitted version.

## Conflict of Interest

The authors declare that the research was conducted in the absence of any commercial or financial relationships that could be construed as a potential conflict of interest.

## Supplementary Material

Supplementary Figure S1 Effect of *Streptomyces rimosus* strains LMG 19352 (b1) and *Rhodococcus sp*. R-43120 (b2) on the growth of *F. poae* 2516 and *F. graminearum* PH-1 combined inoculation on TSA plates. Treatments: pg + w (Inoculation with *F. poae* 2516, *F. graminearum* PH-1 and water); pg + b1 (Inoculation *with F. poae* 2516, *F. graminearum* PH-1 and LMG 19352); p + b2 (Inoculation with *F. poae* 2516, *F. graminearum* PH-1 and R-43120). **(A)** Images taken from day 1 till day 5. **(B)** Results of *F. poae* and *F. graminearum* growth from day 1 till day 5, *n* = 5 (five biological reps per time point). Boxplots indicate the median (horizontal lines), 25th and 75th percentile range (boxes) and up to 1.5 × IQR (whiskers).

Supplementary Figure S2 Effect of *Streptomyces rimosus* strains LMG 19352 (b1) and *Rhodococcus sp*. R-43120 (b2) on the biomass of *F. graminearum* PH-1 and *F. poae* 2516 on wheat ears, respectively. Normalized quantitative relative values (NRQ) of *F. graminearum* PH-1 **(A)** and *F. poae* 2516 **(B)** biomass in wheat ears, *n* = 6 (number of ears per treatment, per pathogen combination and per time point). Boxplots indicate the median (horizontal lines), 25th and 75th percentile range (boxes) and up to 1.5 × IQR (Interquartile Range) (whiskers). Different letters indicate significant differences between treatments (*P* < 0.05). NRQ values were assessed at 4 dai in wheat ears.

Supplementary Figure S3 Expression profile of phenylalanine ammonia-lyase (PAL) at 4 and 7 dai by *F. graminearum* PH-1 (g), *F. poae* 2516 (p) or a combination of both pathogens (pg) and treated with water (+w), with *S. rimosus* LMG 19352 (+b1), with *Rhodococcus sp*. R-43120 (+b2) or with the fungicides prothioconazole + spiroxamine (f). Bars represent log_2_-transformed means of four biological replicates; error bars indicate ± 1 standard error. Different letters indicate significant differences (*P* < 0.05) between treatments for each time point within the same pathogen combination. Fold change was calculated by dividing the CNRQ values (calibrated normalized relative quantities) of the treated ears by values of the control ears. Normalization of defense genes was performed by using the cell division control protein gene (Ta54227) in wheat as reference. The experiment was repeated twice in time.

Supplementary Figure S4 Visual scoring assessment of ears inoculation.

Supplementary Table S1 Primers used for RT-qPCR.
