# Supplementary material for: Presence of the Weakly Pathogenic Fusarium poae in the Fusarium Head Blight Disease Complex Hampers Biocontrol and Chemical Control of the Virulent Fusarium graminearum Pathogen
Source: Front Plant Sci. 2021 Feb 17;12:641890. doi: 10.3389/fpls.2021.641890 (PMC7928387; doi:10.3389/fpls.2021.641890)
Supplement: Supplementary Figure S1 — Effect of Streptomyces rimosus strains LMG 19352 (b1) and Rhodococcus sp. R-43120 (b2) on the growth of F. poae 2516 and F. graminearum PH-1 combined inoculation on TSA plates. Treatments: pg + w (Inoculation with F. poae 2516, F. graminearum PH-1 and water); pg + b1 (Inoculation with F. poae 2516, F. graminearum PH-1 and LMG 19352); p + b2 (Inoculation with F. poae 2516, F. graminearum PH-1 and R-43120). (A) Images taken from day 1 till day 5. (B) Results of F. poae and F. graminearum growth from day 1 till day 5, n = 5 (five biological reps per time point). Boxplots indicate the median (horizontal lines), 25th and 75th percentile range (boxes) and up to 1.5 × IQR (whiskers). [file Data_Sheet_1.docx]

Supplementary Material

# Supplementary Tables

**Table S1** Primers used for RT-qPCR.

| Gene | Forward(5’-3’) | Reverse(5’-3’) |
| --- | --- | --- |
| *FGSG_01244* | TTGGTTGCAGAATGGGCTC | CTCAAACTGGAACCATCGG |
| *FPOA_01282* | TTGGTTGCAGAGTGGGCCA | GCTCAAATTGAAACCACCGA |
| *LOX1* | GGCACGCCATCGAGCAGTACG | TACTGCCCGAAGTTGACCGCC |
| *LOX2* | AACAAGTTCGCCGTCACCTT | TTGTCGAGGGTGATGGTCTT |
| *PAL* | TTGATGAAGCCGAAGCAGGACC | ATGGGGGTGCCTTGGAAGTTGC |
| *ICS* | AGAAATGAGGACGACGAGTTTGAC | CCAAGTAGTGCTGATCTAATCCCAA |
| *Ta54227* | CAAATACGCCATCAGGGAGAACATC | CGCTGCCGAAACCACGAGAC |

# Supplementary figures

**Figure S1** Effect of *Streptomyces rimosus* strains LMG 19352 (b1) and *Rhodococcus sp*. R-43120 (b2) on the growth of *F. poae* 2516 and *F. graminearum* PH-1 combined inoculation on TSA plates. Treatments: pg+w (Inoculation with *F. poae* 2516, *F. graminearum* PH-1 and water); pg+b1 (Inoculation *with F. poae* 2516, *F. graminearum* PH-1 and LMG 19352); p+b2 (Inoculation with *F. poae* 2516, *F. graminearum* PH-1 and R-43120). (a) Images taken from day 1 till day 5. (b), Results of *F. poae* and *F. graminearum* growth from day 1 till day 5, n=5. Boxplots indicate the median (horizontal lines), 25th and 75th percentile range (boxes) and up to 1.5×IQR (whiskers).


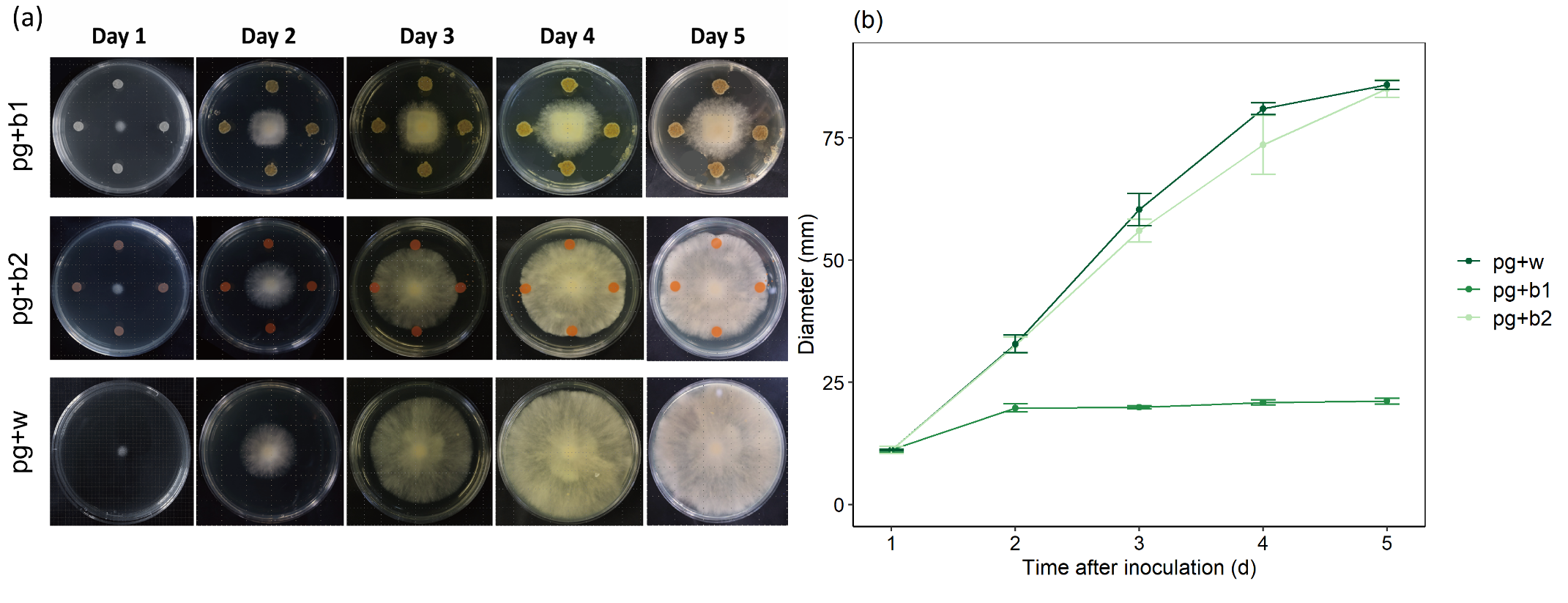


**Figure S2** Effect of *Streptomyces rimosus* strains LMG 19352 (b1) and *Rhodococcus sp*. R-43120 (b2) on the biomass of *F. graminearum* PH-1 and *F. poae* 2516 on wheat ears, respectively. Normalized quantitative relative values (NRQ) of *F. graminearum* PH-1 (a) and *F. poae* 2516 (b) biomass in wheat ears, n=6 (number of ears per treatment, per pathogen combination and per time point). Boxplots indicate the median (horizontal lines), 25th and 75th percentile range (boxes) and up to 1.5×IQR (Interquartile Range) (whiskers). Different letters indicate significant differences between treatments (P<0.05). NRQ values were assessed at 4dai in wheat ears.


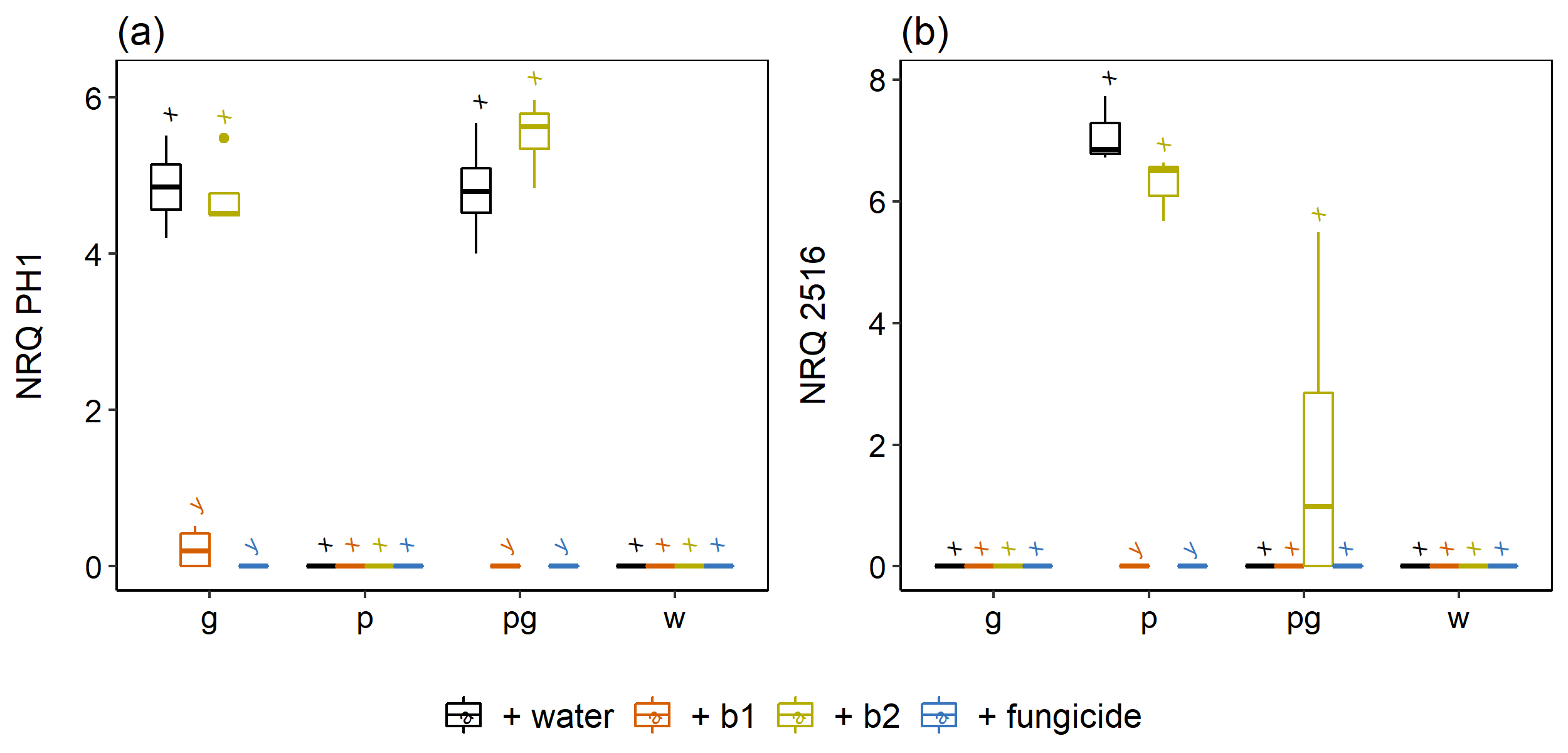


**Figure S3** Expression profile of phenylalanine ammonia-lyase (PAL) at 4 and 7 dai by *F. graminearum* PH-1 (g), *F. poae* 2516 (p) or a combination of both pathogens (pg) and treated with water (+w), with *S. rimosus* LMG 19352 (+b1), with *Rhodococcus sp*. R-43120 (+b2) or with the fungicides prothioconazole + spiroxamine (f). Bars represent log_2_-transformed means of 4 biological replicates; error bars indicate +/- 1 standard error. Different letters indicate significant differences (P<0.05) between treatments for each time point within the same pathogen combination. Fold change was calculated by dividing the CNRQ values (calibrated normalized relative quantities) of the treated ears leaves by values of the control ears. Normalization of defence genes was performed by using the cell division control protein gene (Ta54227) in wheat as reference. The experiment was repeated twice in time.

**
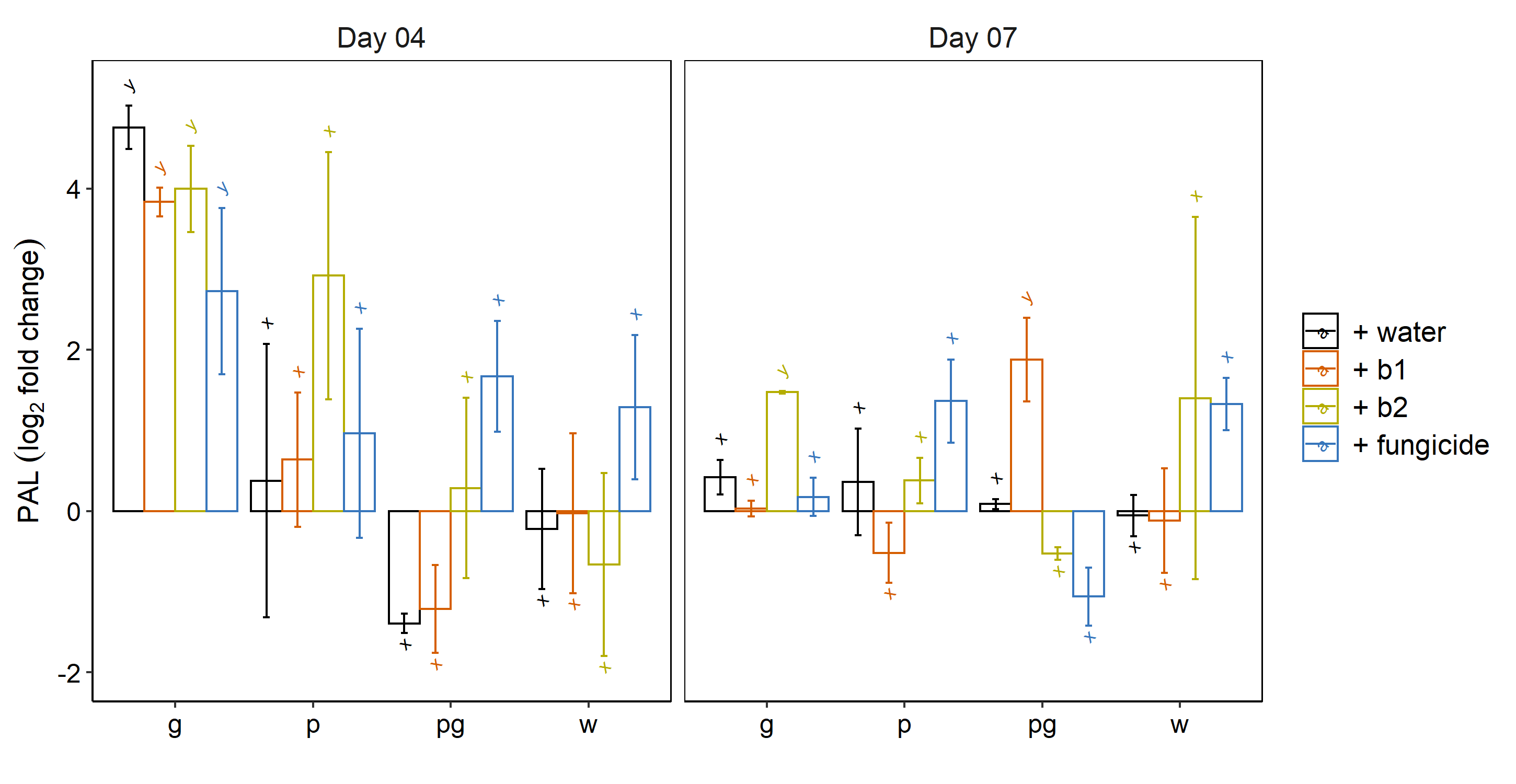
**

**Figure S4** Visual scoring assessment of ears inoculation.

**
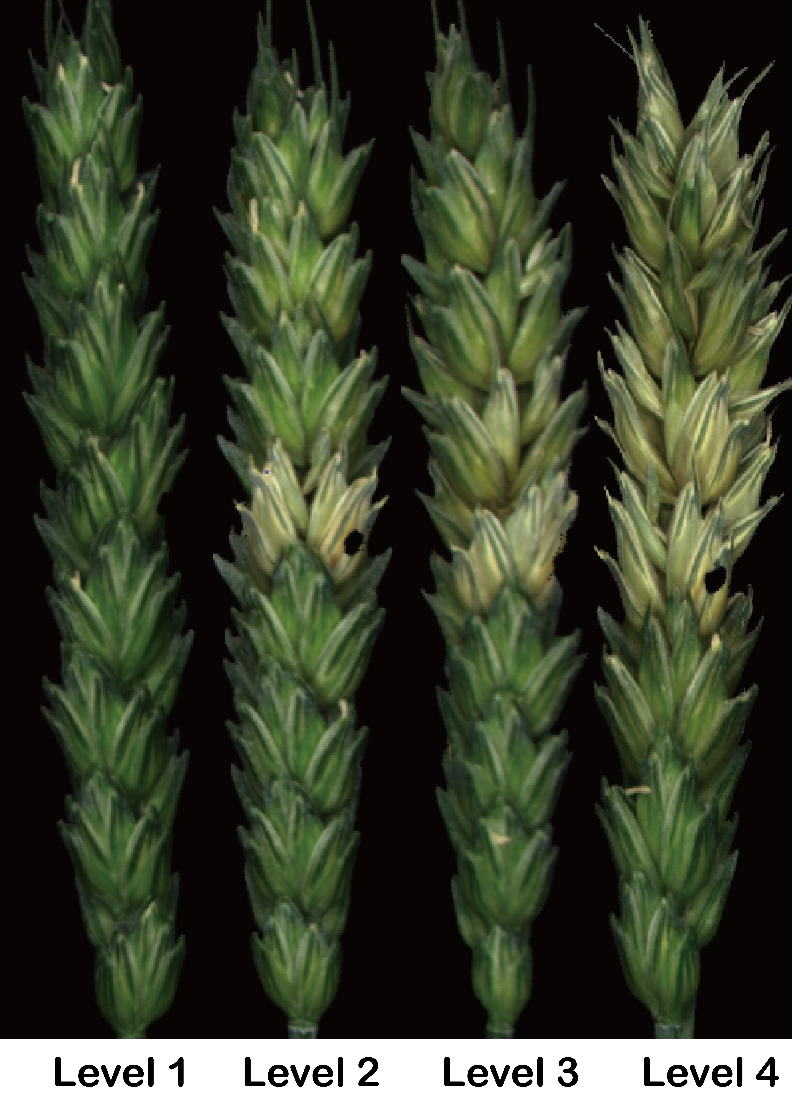
**
